# Authors’ Reply: In the liminal spaces of mental health law – what to do when section 136 expires?

**DOI:** 10.1192/bjb.2024.29

**Published:** 2024-06

**Authors:** Khalil Hassanally, Judy Laing, Anupam Kishore

**Affiliations:** Higher Trainee in Psychiatry, Northwick Park Mental Health Unit, UK. Email: khassanally@nhs.net; Professor of Mental Health Law and Policy, University of Bristol, UK. Email: j.m.laing@bristol.ac.uk; Consultant Psychiatrist, Park Royal Centre for Mental Health, London, UK. Email: anupam.kishore@nhs.net

We thank Dr Memon for his response. Although we agree that this scenario should have been foreseeable by Parliament, the extent to which mental health beds have been cut in England (more than 20% since 2010) may not have been anticipated at the time the amendments to the Mental Health Act were made.^1^

Dr Memon also brings up the important issue of section 140. However, it remains unclear whether providers have yet put adequate policies in place; the last investigation from the Care Quality Commission (CQC) in 2019 suggested that this was not the case.^2^ Furthermore, when writing on section 140, Richard Jones (author of the *Mental Health Act Manual*) states that: ‘this section does not oblige the specified hospitals to admit patients in an emergency or to maintain the capacity to facilitate such admissions, [however] a refusal to admit should only be made with good reason’.^3^

Therefore, in the absence of beds (emergency or otherwise), even knowing the section 140 arrangements may not be enough to resolve the situation. We would be interested to hear whether there are any trusts specifically keeping beds available for the purpose of section 140.

Dr Memon asks what other possible solutions there are. In some areas, the health-based place of safety is converted into a bed, and the patient is detained there. However, as the CQC has pointed out, these so called ‘swing-beds’ can ‘have the effect of worsening the overall situation, by preventing further admissions to the health-based place of safety’.^4^ It is also worth noting that patients are detained in a hospital rather than a bed. For example, a patient who has been brought to an emergency department under section 136 could in theory have admission made out to the hospital that the emergency department is in while a bed at an acute psychiatric facility is located. However, the authors feel that this would rely on an overly literal interpretation of the Act, and, as Richard Jones says: ‘McCullough… in R. v Hallstrom exp. W [1986] Q.B. 1090 [writes] that the term “detention” […] “cannot realistically include a purely nominal period before leave of absence is given, after which the treatment which the patient stands in need is to begin”. Although the decision in Hallstrom was made in the context of a patient who had been granted leave of absence into the community, the finding of McCullough J are equally applicable to a patient who has been granted immediate leave of absence to another hospital. In both cases, the detention at the hospital named in the application would be a sham.’^3^

We would be very interested to hear how other localities address this problem. However, it is worth mentioning that a superficial legal fix will not solve a key concern that Dr Memon brings up: that of moral distress and moral injury that psychiatrists and their colleagues experience when treating patients in suboptimal conditions.
